# 2101: Addressing African American glaucoma through genetics and electronic health records

**DOI:** 10.1017/cts.2017.57

**Published:** 2018-05-10

**Authors:** Jessica Cooke Bailey, Stephanie A. Hagstrom

**Affiliations:** Case Western Reserve University, Cleveland, OH, USA

## Abstract

OBJECTIVES/SPECIFIC AIMS: The overall goal of this project is to understand the genetic and clinical differences in POAG that specifically increase risk in individuals of African genetic ancestry. We will approach this goal by completing the following objectives: (i) localize a genetic signal that accounts for the significantly increased risk for primary open-angle glaucoma in African Americans and (ii) utilize electronic health records (EHR) data to expand our understanding of risk to incorporate endophenotypes of glaucoma and other clinically recorded variables that may influence disease risk. METHODS/STUDY POPULATION: We will genotype at least 200 available African American samples with glaucoma on the Illumina Infinium^®^ Expanded Multi-Ethnic Genotyping Array (MEGAEX) and perform admixture mapping. We will then access EHR data to expand our analysis beyond glaucoma to encompass other relevant risk modifiers captured in the clinical record. RESULTS/ANTICIPATED RESULTS: We anticipate localizing a genetic signal or signals that may account for the increased POAG risk in African Americans. Our calculations indicate that we have ~81% power to detect association at a LOD score of 2 and a risk ratio of 2. Thus, we are well-powered to detect a true signal at this modest level of association DISCUSSION/SIGNIFICANCE OF IMPACT: This project will not only help to achieve precision medicine by filling in the gaps in knowledge regarding glaucoma in African Americans, but it will also address health disparities and aid in the realization of the full potential of “big data” so that all of these elements can be incorporated into a better understanding of health disparities.

